# Behind the Healing: Exploring the Psychological Battles of Kidney Transplant Patients: A Qualitative Insight

**DOI:** 10.1002/hsr2.70511

**Published:** 2025-02-24

**Authors:** Tsiyon Birhanu wube, Solomon Gebremichael Asgedom, Abrehet Girmay Mengesha, Yohannes Ayalew Bekele, Lielt Gebreselassie Gebrekirstos

**Affiliations:** ^1^ Department of Surgical Nursing, School of Nursing College of Health Sciences and Comprehensive Specialized Hospital, Aksum University Axum Ethiopia; ^2^ School of Psychology College of Education and Behavioral Studies, Addis Ababa University Addis Ababa Ethiopia; ^3^ School of Nursing and Midwifery College of Health Science, Addis Ababa University Addis Ababa Ethiopia; ^4^ Department of Maternity and Reproductive Health Nursing College of Health Science and Medicine, Wolaita Sodo University Wolaita Sodo Ethiopia

**Keywords:** experience, kidney, psychological, qualitative, transplantation

## Abstract

**Background and Aims:**

Kidney transplantation is widely regarded as the optimal treatment for end‐stage renal disease, offering benefits like prolonged life expectancy, enhanced quality of life, and greater cost‐effectiveness compared to dialysis. While dialysis imposes considerable restrictions on patients, including diminished independence, many of these challenges can be alleviated through successful transplantation. However, despite the physical advantages of kidney transplants, the psychological struggles faced by transplant recipients are often overlooked, particularly in Ethiopia. This study aimed to delve into the psychological experiences of kidney transplant patients, shedding light on the emotional and mental battles they encounter post‐transplant.

**Methods:**

This qualitative study involved kidney transplant patients at SPMMC (St. Paul's Millennium Medical College). during the study period, regardless of donor type. A total of ten participants were selected using purposive sampling. Data were collected through semi‐structured, interviewer‐administered questionnaires containing both closed and open‐ended questions. The data were transcribed verbatim and analyzed using interpretative phenomenological analysis (IPA). The analysis focused on understanding participants' experiences and their interpretations of these experiences. Themes were identified by examining commonalities and differences in the participants' accounts, with special attention to areas of convergence and divergence.

**Results:**

The analysis revealed both positive and negative psychological experiences among kidney transplant patients. Positive experiences included feelings of “re‐birth,” thankfulness, strengthened social bonds, freedom from dialysis, enhanced self‐efficacy, and increased social support. Negative experiences included dependency, fear of the future, challenges with adherence to treatment, and occasional regret.

**Conclusion:**

Life after a kidney transplant is significantly more positive and empowering compared to life on dialysis. However, psychological challenges remain, highlighting the need for ongoing psychological support to address both positive and negative aspects of the transplant experience. Future research should focus on providing continuous support, including counseling and support groups, and integrating these systems into kidney transplant care to enhance overall recovery.

## 1 Introduction

Chronic kidney disease (CKD) has emerged as a critical public health challenge globally, affecting approximately 10.4% of men and 11.8% of women [1]. The consequences of CKD extend far beyond impaired kidney function; they encompass a spectrum of emotional and psychological challenges that profoundly impact patients' lives. With an estimated 5.3 to 10.5 million individuals requiring dialysis or transplantation, the burden of CKD is immense, yet many patients face obstacles such as financial constraints and lack of resources that hinder access to essential treatments [2, 3].

Among the treatment options, renal transplantation is often regarded as the most favorable, promising enhanced longevity and a better quality of life compared to dialysis [4, 5]. While the transition from dialysis to transplantation can be a source of renewed hope, it is also accompanied by significant emotional upheaval [6, 7]. Patients frequently experience a profound shift in identity and a complex array of feelings that can include gratitude towards the donor, joy at newfound independence, and anxiety about their health status and future [8, 9]. CKD and dialysis are highly restrictive for patients due to several factors. The treatment requires patients to adhere to a strict medication regimen to manage complications such as hypertension, anemia, and electrolyte imbalances. Additionally, dialysis sessions are time‐consuming, 2 often taking several hours, three times a week, which disrupts daily activities and limits patients' ability to engage in normal life routines [10]. The physical discomfort associated with dialysis, including pain, fatigue, and the general toll on the body, further exacerbates the challenge. On top of this, patients must follow strict dietary and fluid restrictions to avoid complications, which can be difficult to manage and impact their quality of life [11]. These combined restrictions contribute to the significant burden dialysis places on patients, highlighting the positive impact that kidney transplantation can have in offering greater freedom and improved well‐being [12].

Despite the benefits of transplantation, the psychological ramifications of this life‐altering procedure are frequently overlooked. Research indicates that while kidney transplant recipients often report feelings of liberation and gratitude, they also contend with negative psychological outcomes such as depression, anxiety, and fear [13, 14]. The journey toward psychological adjustment post‐transplant is multifaceted, influenced by factors such as acceptance of the new organ, medication side effects, and the ever‐present anxiety about organ rejection [15, 16].

Ethiopia, in particular, faces a critical gap in understanding the psychological experiences of kidney transplant patients. Despite the increasing prevalence of CKD and renal transplantation in the region, there is a notable absence of empirical research focusing on the mental health challenges faced by these patients [17, 18, 19]. As CKD continues to be significantly more common in developing countries compared to their developed counterparts, it is imperative to explore the emotional landscape of those navigating the complexities of kidney transplantation [20, 21].

This study aims to bridge this gap by providing qualitative insights into the psychological battles faced by kidney transplant patients. By delving into the lived experiences of these individuals, we hope to highlight the intricate interplay between psychological health and transplant outcomes. Understanding the emotional and psychological dimensions of the transplant experience is not only vital for improving patient care but also essential for fostering a holistic approach to health that integrates mental well‐being into the recovery process.

## 2 Methods

### 2.1 Study Design

A qualitative research approach using Interpretative phenomenological analysis (IPA) and purposive sampling was employed. IPA was chosen to explore participants' lived experiences and the meanings they attribute to specific events. This method is ideal for capturing both individual perceptions and shared experiences, providing an insider's perspective on the study topic. IPA helps uncover how participants make sense of their experiences, focusing on personal understanding rather than broad generalizations, and offers various approaches for qualitative data interpretation and analysis.

### 2.2 Study Area and Period

The study was conducted at St. Paul's Millennium Medical College (SPMMC) in Addis Ababa, Ethiopia, among kidney transplant patients from May 15 to May 20, 2022. SPMMC is a 350‐bed hospital located in Addis Ababa, serving a catchment population of 5 million. The first Ethiopian kidney transplant program was launched at SPMMC in September 2015 in collaboration with the University of Michigan. The transplant center includes three nephrologists, four transplant surgeons, 20 nurses, and additional support staff, with a functioning 15‐bed dialysis unit, a four‐bed intensive care unit (ICU), and a 24‐h pharmacy and laboratory. A total of 1,800 patients with chronic kidney disease (CKD) were on the transplant waiting list, and 350 kidney transplant patients are currently under follow‐up at the hospital, with 150 having received their transplants there.

### 2.3 Participants

All clients diagnosed with chronic renal failure were considered for this study. The study population consisted of kidney transplant patients, regardless of the type of organ donor, who were available during the study period at St. Paul's Millennium Medical College.

### 2.4 Personal Characteristics of Researcher

The interviews were conducted by TBW (first author), a female researcher with a Master's degree in health psychology and formal training in qualitative research methods. TBW's background in health psychology and experience in conducting qualitative research ensured that she was well‐equipped to explore the complex psychological experiences of kidney transplant recipients. Her expertise in conducting semi‐structured interviews allowed her to guide participants through in‐depth discussions while maintaining an empathetic and non‐judgmental approach.

### 2.5 Sample and Sampling

IPA typically requires a small sample size to provide a detailed understanding of participants' experiences and perspectives. A total of ten participants were selected using purposive sampling. Specifically, the sample included patients who had undergone transplantation and were receiving follow‐up care during the study period. Participants were recruited using a purposive sampling method, aiming to select individuals capable of providing in‐depth insights into the psychological experiences associated with transplantation. Recruitment was guided by predefined inclusion criteria, including the participants' willingness to take part in the study and their ability to articulate their experiences relevant to the research objectives. To address potential selection bias, several measures were implemented. First, clear and transparent inclusion criteria were consistently applied to ensure fairness in participant selection. Second, recruitment decisions were guided strictly by these criteria, minimizing the influence of subjective judgments by the research team. Third, the entire recruitment process was systematically documented to maintain transparency and accountability. These steps ensured that the selection process was rigorous and aligned with the study's qualitative research design.

## 3 Eligibility

### 3.1 Inclusion Criteria

Kidney transplant patients aged 18 and older, regardless of the type of donor, who had follow‐up care at St. Paul's Millennium Medical College were included in the study.

### 3.2 Exclusion Criteria

Patients who had undergone re‐transplantation, were critically ill, clinically unstable, or mentally impaired during the data collection period were excluded from the study.

### 3.3 Data Collection Tools

Semi‐structured interviews were used to collect data, consisting of closed and open‐ended questions. The tool comprised three sections [1]: patients' clinical and socio‐demographic data, and [2] three key questions: “What does having a kidney transplant mean to you?”, “What are the main psychological changes following the transplant?”, and “What are the principal difficulties faced post‐transplant?”. Interviews were conducted by the data collector. The tool was initially developed in English, translated into Amharic by a certified translator, and then back‐translated into English to ensure consistency and clarity.

### 3.4 Personal Characteristics

The interviews were conducted by LGG, a female researcher with a Master's degree in health psychology and formal training in qualitative research methods. TBW's background in health psychology and experience in conducting qualitative research ensured that she was well‐equipped to explore the complex psychological experiences of kidney transplant recipients. Her expertise in conducting semi‐structured interviews allowed her to guide participants through in‐depth discussions while maintaining an empathetic and non‐judgmental approach.

### 3.5 Data Collection Procedure

In‐depth interviews were recorded electronically. Participants were invited to take part during their routine follow‐up consultations. The interviews were conducted individually on the same day as the consultations. Participants were fully informed about the study's aims, and the interviews were held in private settings to encourage free expression. The interviewers addressed any concerns participants had, allowing them to discuss topics in depth. The interviews were conducted by a researcher with formal training in health psychology and qualitative research methods, ensuring expertise in guiding in‐depth interviews and understanding complex psychological themes. The interviewer had no prior clinical or medical care engagement with the participants, ensuring neutrality and minimizing bias during the data collection process. Specifically, the interviews were conducted by the primary data collector, a trained researcher with a Master's degree and expertise in qualitative data collection. Their role involved administering the semi‐structured questionnaires, creating a comfortable environment for participants, clarifying the purpose of the study, facilitating the discussions, asking relevant questions, and accurately recording responses. On average, each interview lasted approximately 45 to 60 min, allowing sufficient time for participants to share their experiences in detail while maintaining focus on the research objectives.

No repeat interviews were conducted in this study. Each participant was interviewed once. Audio recordings were used to collect the data, which were later transcribed for analysis. To complement the recordings, field notes were taken by the researcher after each interview to capture any additional observations or reflections that might not have been recorded. Data saturation was discussed, and it was determined that saturation was reached when no new themes or insights emerged from the subsequent interviews. Transcripts were not returned to participants for comment, although they were given the opportunity to ask any clarifying questions or provide additional comments after the interviews if they wished.

### 3.6 Data Quality Management

Verbatim transcription of the interview data was conducted. The transcriptions were read multiple times to identify key ideas and themes. Audio recordings were also reviewed multiple times without note‐taking to capture the overall sense of the conversations and ensure accuracy.

### 3.7 Relationship with Participants

Before the study, no relationship had been established between data collector and the participants, as she had no direct clinical or medical care involvement with them. Participants were fully informed about TBW's academic background and the purpose of the research, but they were assured that their participation would remain confidential and separate from any healthcare decisions. TBW's neutral stance during the interviews fostered an environment of trust and openness, enabling participants to share their experiences candidly. Her approach was designed to ensure that participants felt comfortable discussing both the positive and challenging aspects of their transplant journey without feeling influenced by any pre‐existing relationships or biases.

### 3.8 Data Analysis

Data were analyzed using IPA. The interviews were transcribed and analyzed according to established IPA guidelines. The analysis focused on understanding participants' experiences and heir interpretations of these experiences, while suspending any prior assumptions about the topic. Each transcript was reviewed to identify emerging themes, which were then compared across participants to explore patterns of convergence and divergence. Themes were grouped, and final titles were assigned to reflect the depth and scope of the data. This process resulted in a narrative structure that encapsulated the main themes across the participants. For the data analysis in this study, a single researcher, the primary data collector, coded the data independently. The coding process was inductive, with themes and subthemes emerging directly from the data. A coding tree was developed through an iterative process, where recurring patterns and significant concepts were identified across the data set. The researcher used NVivo software to manage, organize, and facilitate the coding and analysis of the qualitative data. Themes were derived from the participants' responses, and no pre‐defined themes were used. Participants were not asked to provide feedback on the findings, and the analysis relied solely on the in‐depth review of the interview data without any member checking being conducted.

### 3.9 Ethical Considerations

Ethical approval was obtained, and a formal letter of cooperation was submitted to St. Paul's Millennium Medical College. Written informed consent was obtained from all participants before the interviews, after they were fully informed about the study's objectives. Participants were assured that they could withdraw from the study at any time without consequence. Privacy was protected, and no identifying details were recorded or reported in any study documents. Participation was entirely voluntary.

## 4 Results

This chapter presents the findings in line with the study's objectives. In this qualitative study, a total of 10 in‐depth interviews with kidney transplant patients were conducted. Respondents' ages ranged from 28 to 56 years. All the donations were from living donors. Of the total respondents, more than two‐thirds were male, and all were literate, having had their transplants for two or more years (Table 1).

**Table 1:** Socio‐demographic characteristics of in‐depth interview participants, St. Paul Hospital, Addis Ababa, Ethiopia, 2022.

Age	Sex	Marital status	Educational status	Relationship with the donor	Duration since transplantation	Average monthly income
29	M	Married	Secondary	Wife	24 months	18000
28	F	Married	Primary	Uncle	28 months	Prefer not to tell
38	M	Married	Secondary	Wife	24 months	5000
52	M	Married	Above Secondary	Brother	24 months	17000
48	M	Married	Above Secondary	Brother	24 months	15000
30	M	Married	Above Secondary	Father	25 months	20000
38	F	Single	Above Secondary	Father	36 months	14000
37	M	Single	Above Secondary	Sister	33 months	None
56	M	Single	Above Secondary	Brother	42 months	16000
28	F	Single	Above Secondary	Brother	36 months	None

Under the two main themes of this study, the following sub‐themes emerged: transplantation as a re‐birth, thankfulness, social bond, liberty from dialysis, self‐efficacy, and social support were identified as positive psychosocial experiences, while dependency, fear of the future, adherence challenges, and regret emerged as negative psychosocial experiences.

### 4.1 Affective Experience of Participants During Interviews

Participants displayed a wide range of emotions during the interviews. Many expressed gratitude for the opportunity to share their experiences, describing the process as therapeutic and reflective. While some shared their stories with optimism and enthusiasm, others displayed moments of vulnerability, particularly when discussing challenges or negative emotions associated with their transplant journey. The interviewer maintained a supportive and empathetic environment to encourage open and honest communication throughout the sessions.

### 4.2 Positive Psychosocial Experiences

#### 4.2.1 Kidney Transplantation as a Re‐Birth

Most kidney transplant clients described transplantation as a process of new life and revival. A 52‐year‐old university professor explained that he felt he had restarted life again, considering the life he lives now as extra years of life: *“Kidney transplantation is a means to resume life; it is like restarting life… There are many people who just pass away in their sleep. As for me, I am now living an extra life from the day my kidney was transplanted, because the day my kidney failed, I thought it was my last day on earth, and now I am living extra years and very happy with it.”*

Another 38‐year‐old man echoed this sentiment, stating that his life was renewed after the transplantation: *“I am now like a child, like a newborn. I frankly tell you; I feel as if I did not transplant, rather as a newborn. I'm living a very peaceful life.”*

#### 4.2.2 Thankfulness

Participants expressed deep gratitude toward donors, health professionals, and all forms of support they received. A university lecturer explained: *“I had a lot of psychological support from all sides. People around me tried their best to make it as simple as possible. They said, ‘the current technology is implanting a heart from the dead, so your kidney is very simple.’ I had tremendous support of all kinds, from my students, the community, and all staff.”*

All participants expressed their gratitude to the health professionals at St. Paul Hospital. One interviewee said: *“They [health professionals] are very helpful. They tried their best. They are people in need and amazing. I am grateful forever.”*

#### 4.2.3 Liberty From Dialysis

All participants who underwent transplantation mentioned they were freed from the pain, suffering, and financial burden caused by dialysis. A 28‐year‐old woman who received a kidney from her uncle said: *“It is so different now. I came back to life after transplantation, but during the dialysis, I had countless problems like pain, income challenges, and many others. But now, I am living like any normal person.”*

Another interviewee who received a kidney from his brother explained that life with a transplanted kidney was like a resurrection from the harsh and unlivable conditions experienced during dialysis: *“Immediately after transplantation, I saw a radical change in my life. I called it resurrection. During the dialysis time, whatever symptoms appeared, I felt like I was dying and suffered a lot. But after that [transplantation], all my body functions returned to normal. It has been 4 years and 6 months now. I am very healthy and leading a normal life. I am a free man.”*

A 56‐year‐old man reaffirmed this, saying: *“I never thought human beings could tolerate such painful times, especially during dialysis. I had horrible nights at the dialysis center, which is now history. I am grateful that transplantation resolves all problems.”*

#### 4.2.4 Self‐Efficacy

Participants expressed confidence in their ability to accomplish tasks, which was not the case before transplantation. One interviewee stated that he is now able to do the same work as before his illness, saying: *“I've never felt or thought that I am missing something or weaker than anyone else. I have been informed not to handle heavy materials and not to do home tasks, which I am not doing. However, if I had to do something, I think I would be able to. I am a lecturer, and I'm giving lectures as before and as equal to other academic staff. Going back to work has a lot of meaning to me. I am competent enough.”*

Another 38‐year‐old woman mentioned how things changed for the better after transplantation: *“Before transplantation, I was not able to do any type of work. But after transplantation, I am able to work and eat what I desire, as per the recommendation. I was 51 kg, but now I am 61 kg. I am able to eat the foods I want.”*

#### 4.2.5 Social Support and Social Bond

Participants explained that the social support they received from family, friends, and others played a significant role in their self‐image and recovery. One participant said: *“I have learned the vitality of one to another. It is because of the support, in all ways—financially, psychologically, and otherwise—that I am alive today. People can build your morale and enable you. I also got support from people I never expected, which astonished me and changed me forever.”*

Another participant discussed the role of social support in the healing process, saying: *“Our communities care very much and try their best to support us in times of need. After transplantation, there was a lot of support from friends, co‐workers, and family, which had a positive influence on my healing process.”*

#### 4.2.6 Adherence

Participants explained that maintaining their health status and quality of life through prescribed medication was paramount, though it can be difficult to follow. One participant said: *“I have some concerns with the medications I'm taking, as it is lifelong. I feel bored by all the precautions I should take, including taking medications on time. I don't know how long I will carry on, but I thank God and strictly adhere to it.”*

### 4.3 Negative Psychosocial Experiences

#### 4.3.1 Fear of the Future

Almost all participants expressed concern and worry about the future. They shared uncertainties about what would happen to them and their donors. A recipient who received a kidney from his wife described his fear, particularly in the early days after the transplant: *“I was terrified after the transplantation, thinking, ‘what if my wife and I die? What if the transplantation fails? What if my wife dies, who gave me her kidney? Who will raise my children?’ Those were questions I could not answer, and they terrified me in the early weeks after the transplant. But as time passed, things returned to normal.”* Participants did express concerns about the potential return to dialysis. Many participants feared that if their transplanted kidney failed, they might have to undergo dialysis again. However, the issue of not qualifying for another transplant was not explicitly mentioned by the participants.

Another female participant who received a kidney from her father expressed worry about her father's health: *“I always fear that if I suddenly die, I imagine how my dad would feel. I also worry about his health—what if he develops complications from the donation? I always fear for the future.”*

Another participant, who received a kidney from his wife, shared his fear: *“I got the kidney from my wife and always worry about her and what will happen to us. We both have the same fate—kidney failure. That troubles me, and I feel so stressed about it. When I hear about someone facing failure after a few years of transplantation, I get scared, wondering if that pain will return to me. That fear and uncertainty about the future causes stress.”*

### 4.4 Dependency

Another Subtheme that emerged relates to the feeling of dependency, which developed due to how others treat the patients and perceive them. A participant explained his experience and how his family views him: *“Sometimes when my family members restrain me from doing anything at home, I feel as if I am not able to contribute, but they are taking care of me, which reassures me though.”* [IDI 5]. The participants highlight the emotional and practical challenges of not being able to work full‐time and rely financially on others.

Another interviewee expressed that he no longer plays the significant social role he once did. He shared his experience: *“I once saw people fighting, which could have possibly harmed me. I just restrained myself, which was not the case before. I used to go and mediate, but now all of a sudden, I think about the person who gave me their kidney. So, I restrained myself.”* [IDI 1]

### 4.5 Feeling of Peculiarity

Another negative experience that emerged from the data was the feeling of being different. A participant explained how this feeling of peculiarity changed his entire way of life, making him feel as though he is always on standby:

One participant described how the feeling of being different impacted both his physical and social life: *“Before the transplantation, I used to lead a simple life, but not anymore. I am different now. I have totally changed my way of life, so restricted in many ways. I don't go out or socialize like I used to. I feel as though I'm always on alert, unable to live like others. My day‐to‐day life feels like it's on hold.”* [IDI 10]. This reflects a sense of social isolation and constant vigilance, both physically and socially.

Another participant shared their concern about having a transplanted kidney, which caused them to feel out of place and always worried about their health: *“The kidney I have now is not mine, which makes me constantly worry about it. Before, I never had these concerns and just took life as it came. Now, I have to be extra careful in everything I do. It's like living with a constant reminder that I'm different.”* [IDI 5]. This highlights the ongoing anxiety and sense of difference in terms of both physical condition and the social aspects of life.

### 4.6 Regret

Another Subtheme that emerged from the data was regret over the decision to receive a kidney from a family member. One participant who received a kidney from his wife expressed his sadness and guilt over the situation, saying: *“Sometimes I feel I should not have done it [received the donation]. She would have lived like anyone else if she had not donated to me. It brings a lot of pressure.”* [IDI 3] (Table 2).

**Table 2:** Table of quotations for exploring the psychological battles of kidney transplant patients.

Theme	Subtheme	Quotation	Participant ID
Positive experiences	Rebirth	“Kidney transplantation is a means to resume life; it is like restarting life… now I am living extra years and very happy with it.”	IDI 1
	Thankfulness	“I had tremendous support of all kinds, from my students, the community, and all staff.”	IDI 3
	Freedom from dialysis	“It is so different now. I came back to life after transplantation, but during dialysis, I had countless problems.”	IDI 5
	Self‐efficacy	“I feel competent enough… I'm giving lectures as before and as equal to other academic staff.”	IDI 7
	Social support and bonds	“It is because of the support… financially, psychologically, and otherwise, that I am alive today.”	IDI 8
Negative experiences	Fear of the future	“What if the transplantation fails? What if my wife dies, who gave me her kidney? Who will raise my children?”	IDI 4
	Dependency	“Sometimes when my family members restrain me… I feel as if I am not able to contribute.”	IDI 5
	Feeling of peculiarity	“I feel as though I'm always on alert, unable to live like others. My day‐to‐day life feels like it's on hold.”	IDI 10
	Regret	“Sometimes I feel I should not have done it [received the donation]. She would have lived like anyone else if she had not donated to me.”	IDI 3

### 4.7 Thematic Schema

#### 4.7.1 Major Themes and Sub‐Themes

**Figure 1:**
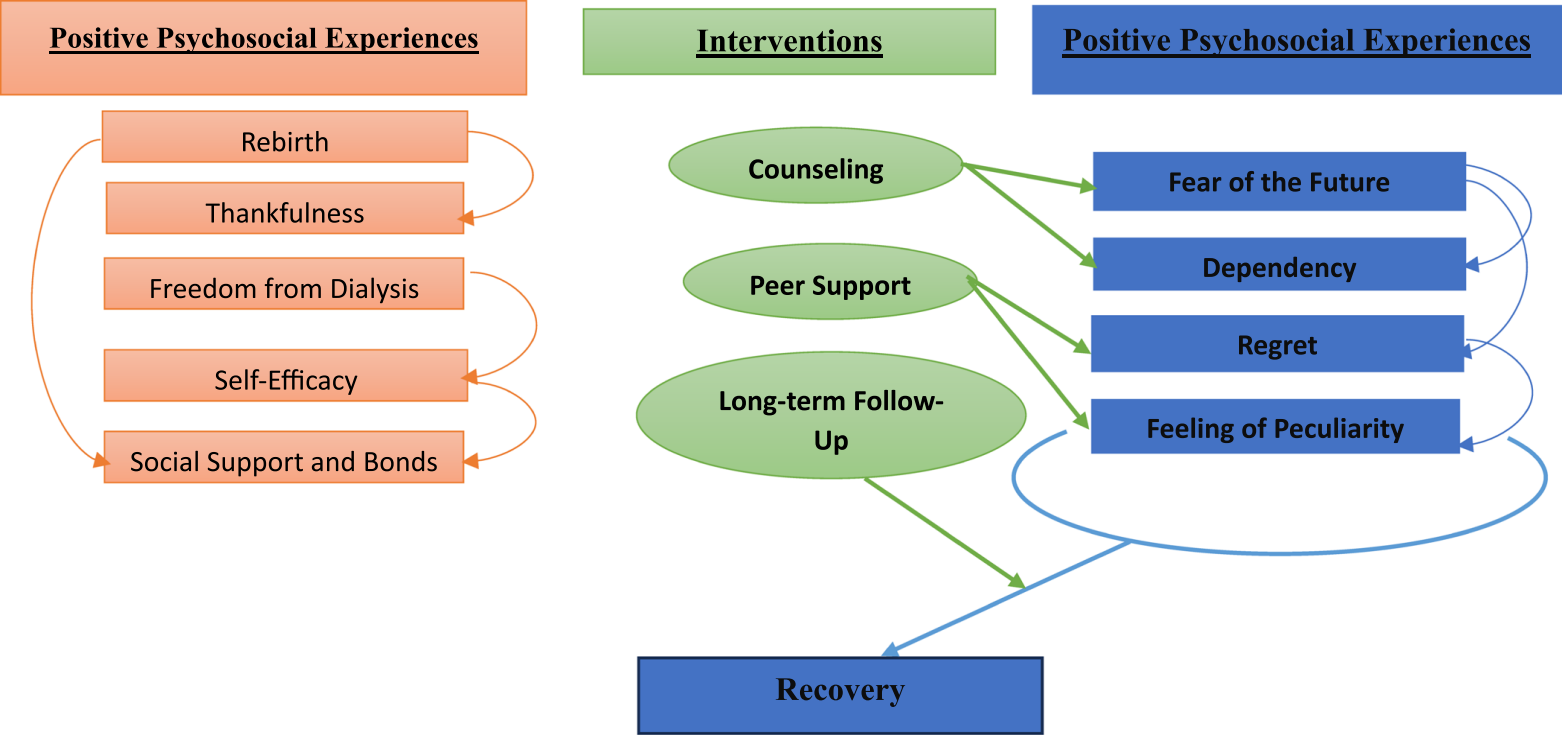
A conceptual graph showing the interconnections among major themes (positive and negative psychosocial experiences, interventions), sub‐themes, and their relationships.

The interconnected themes reveal how Positive Psychosocial Experiences, like rebirth, thankfulness, social support, and freedom from dialysis, foster self‐efficacy and renewed hope, while Negative Psychosocial Experiences, such as fear of the future, dependency, regret, and feeling of peculiarity, highlight emotional and social challenges. Interventions and Future Plans, including counseling, peer support, family‐centered care, and long‐term follow‐up, address these challenges by reducing anxiety, fostering connection, and supporting sustained recovery, emphasizing the importance of holistic care in enhancing overall well‐being (Figure 1).

## 5 Discussion

Participants' reports highlighted diverse psychological experiences post‐transplant, including feelings of gratitude, anxiety about graft survival, and the pressure to maintain health to honor their donor's sacrifice. They also revealed challenges in adapting to lifestyle changes, such as strict medication regimens and fear of rejection. Additionally, participants noted shifts in social dynamics, particularly within family relationships, emphasizing the multifaceted nature of their post‐transplant journey. Participants from urban areas often expressed greater access to social support and healthcare resources, which contributed to more positive psychological experiences. In contrast, those from rural or remote areas mentioned challenges related to limited access to healthcare services, which impacted their emotional well‐being. This is consistent with previous research [22], who found that access to support networks and healthcare resources plays a critical role in transplant recipients' psychological adjustment. Additionally, cultural factors and family dynamics were significant in shaping the transplant experience. Some participants highlighted strong family support, with donations often coming from close relatives, which positively influenced their psychological well‐being. However, the sense of dependency and guilt associated with receiving a kidney from a loved one was particularly pronounced among those with close familial ties. These findings align with studies [23], which discuss how the emotional burden of transplant recipients can be influenced by family roles and cultural expectations. On the other hand, participants who reported less family involvement or support experienced greater isolation and anxiety about their future, which mirrors findings [24, 25], who noted that lack of support exacerbates negative psychological outcomes.

All participants in this study had been transplanted within the last 2–4 years, which may influence the psychological and emotional experiences they shared. Compared to newly transplanted patients, these individuals might have had more time to adapt to life post‐transplant, develop coping mechanisms, and establish new routines, potentially leading to a more reflective and stable perspective. Conversely, their experiences might differ significantly from patients further out from transplantation, who could face different long‐term challenges, such as chronic complications or graft‐related anxiety.

Regarding the positive psychological experiences, interviewees described transplantation as a “rebirth,” a powerful metaphor that signifies not only the physical act of receiving a new kidney but also a profound transformation in their outlook on life. This aligns with studies such as Lovera [26], which suggest that recipients often experience hope and a renewed desire for life through the act of donation. Many participants expressed a newfound appreciation for life and a stronger desire to engage in meaningful activities and relationships post‐transplant. To further support this transformation, research by Zhao [27] emphasizes the importance of structured psychological interventions, such as counseling and peer support groups, in helping recipients maintain a positive outlook and cope with post‐transplant challenges. These interventions could be integrated into transplant care to foster psychological well‐being alongside physical recovery.

A notable positive experience in this study was the expression of thankfulness. Participants frequently expressed deep gratitude toward their higher power, donors, and support systems for helping them navigate the challenges of transplantation. This sense of gratitude served as a psychological coping mechanism, fostering resilience and promoting emotional well‐being. Similar findings have been reported in studies such as Sieverdes [28] and Cazauvieilh [29], where gratitude toward donors was shown to strengthen social connections and improve mental health outcomes. To build on these findings, interventions such as structured counseling and peer support groups, as proposed by Diniz [30], could enhance the psychological benefits of gratitude by helping recipients process emotions and address potential feelings of guilt or anxiety. Future research should focus on integrating such interventions into transplant care, examining their long‐term impact on emotional resilience, social reintegration, and overall quality of life for kidney transplant recipients.

Liberation from dialysis emerged as a frequently cited positive experience, with recipients describing their pre‐transplant lives as restrictive and oppressive. Many likened transplantation to a form of emancipation, regaining autonomy and freedom from the physical, financial, and emotional burdens of dialysis. This perspective aligns with studies like Aufhauser [31] and Scheuermann [32], which highlight the transformative impact of transplantation on recipients' independence. Participants in this study often reported a renewed sense of empowerment and agency, reclaiming aspects of their lives previously compromised. Interventions such as structured psychosocial rehabilitation programs could further enhance this transformation by helping recipients address lingering emotional challenges and fully embrace their independence. Future research should explore long‐term rehabilitation strategies, including peer mentoring and community reintegration programs, to maximize post‐transplant quality of life. Additionally, upcoming studies, like those mentioned in study [33], emphasize the integration of psychological support into routine transplant care to ensure both physical and emotional recovery. Expanding these interventions could foster a deeper, sustained transformation for recipients.

Fear of organ failure was a significant concern among kidney transplant recipients, often overshadowing positive experiences and leading to heightened anxiety and stress. This aligns with findings from Wurm [33] and Forsberg [16], which identify fear of graft failure as a major psychological challenge. To address this, interventions such as regular mental health screenings, tailored counseling sessions, and peer support groups could provide recipients with the tools to manage anxiety and build resilience. Additionally, education programs that offer clear guidance on the likelihood and management of organ failure can help reduce uncertainty and empower patients. Research by De Pasquale [34] emphasizes that integrating psychological support into routine post‐transplant care significantly improves mental health outcomes and quality of life. Future research should prioritize developing comprehensive care models that include these elements to better support recipients in managing their fears and enhancing their overall recovery experience.

Feelings of dependency and regret emerged as significant negative experiences among kidney transplant recipients. Participants often expressed guilt about potentially endangering their donor's life, leading to moral conflict and emotional turmoil. This aligns with Holscher [35], who highlight similar struggles in the context of altruistic organ donation. Some recipients shared how others' perceptions of their abilities exacerbated feelings of dependency, creating a dual struggle between seeking support and maintaining autonomy. Additionally, regret over imposing burdens on donors was a recurring sentiment, echoing finding by [36], who noted that such emotions can complicate post‐transplant recovery. To address these challenges, interventions should include targeted counseling to help recipients process feelings of guilt and dependency, fostering emotional resilience. Peer support programs, as suggested by Ghahramani [37], could enable recipients to share experiences and strategies for managing these emotions. Practical family‐centered interventions may also alleviate tension by improving communication and shared understanding between recipients and their donors. Future research should explore culturally sensitive approaches to dependency and guilt, particularly in regions where family roles heavily influence the donation process. Upcoming studies could evaluate the effectiveness of ongoing psychological support and structured rehabilitation programs in mitigating these challenges, ensuring holistic care for kidney transplant recipients.

## 6 Strengths and Limitations

This study has several notable strengths. It provides valuable insights into the social and psychological experiences of kidney transplant recipients, highlighting both positive and negative aspects. By exploring these experiences in‐depth, the study contributes to a more comprehensive understanding of the post‐transplant journey, which is often underreported. The relatively small sample size and lack of diversity among participants limit the generalizability of the findings. Additionally, the data were collected from a single setting, which may restrict the external validity of the results. One notable strength of IPA is its ability to provide an in‐depth exploration of participants' lived experiences, capturing rich and nuanced insights into their narratives and psychological themes. This makes it particularly valuable for understanding complex phenomena, such as the emotional and psychological impacts of transplantation. However, a key limitation of IPA is its reliance on small sample sizes, which may restrict the generalizability of findings. Additionally, the method is resource‐intensive, requiring substantial time and expertise for data collection and analysis, which can pose challenges for broader applicability.

Future research should include larger and more diverse samples from multiple healthcare institutions, encompassing a broader range of cultural, rural, and family contexts to enhance the generalizability of the findings. Furthermore, while this study offers in‐depth insights into recipients' experiences, it primarily relies on a qualitative approach. Incorporating standardized assessment tools or validated scales could provide a more objective measure of psychological outcomes, strengthening the reliability and comparability of the results.

## 7 Conclusion

The findings from this study highlight positive and negative aspects of their post‐transplant journey. On the positive side, recipients frequently described feelings of rebirth, gratitude, liberation from dialysis, and enhanced self‐efficacy, all of which contributed to an improved quality of life. However, these positive experiences were often tempered by significant challenges, including fear of organ failure, dependency, and regret over the impact of their decision on donors. The emotional complexity surrounding kidney transplantation calls for a comprehensive approach to patient care that addresses both physical recovery and mental well‐being.

To ensure better long‐term outcomes, healthcare providers should focus on providing tailored psychosocial support to address the fears and emotional burdens associated with organ transplantation. Additionally, structured post‐transplant counseling and support groups could help recipients navigate feelings of dependency and regret, while fostering a positive outlook on their new lives. Encouraging open communication with family members and donors could also alleviate the sense of guilt, creating a more supportive environment for the patient's emotional recovery.

## Author Contributions

**Tsiyon Birhanu Wube:** conceptualization, investigation, funding acquisition, writing–original draft, writing–review and editing, data curation, resources, project administration, validation, methodology, formal analysis. **Solomon Gebremichael Asgedom:** conceptualization, investigation, writing–original draft, writing–review and editing, project administration, formal analysis, resources. **Abrehet Girmay Mengesha:** conceptualization, funding acquisition, writing–original draft, writing–review and editing, project administration, software. **Yohannes Ayalew Bekele:** conceptualization, investigation, writing–original draft, writing–review and editing, data curation. **Lielt Gebreselassie Gebrekirstos:** writing–original draft, funding acquisition, conceptualization, investigation, writing–review and editing, project administration, supervision, data curation.

## Ethics Statement

The study was evaluated and approved by the Institutional Review Board of St. Paul's Millennium Medical College. Written informed consent was obtained from the study participants before the interviews, after fully informing them about the study's aim. All participants were made aware of their right to withdraw from the study at any time without consequence. Privacy was guaranteed, and no identifying personal details were recorded or included in any documentation related to the study. Participation was entirely voluntary, and no one was obligated to take part unless they explicitly agreed.

## Conflicts of Interest

We declare that no funding sources or financial relationships had any involvement in the study design, data collection, analysis, or interpretation, the writing of the report, or the decision to submit the report for publication.

## Supporting information

Supporting information.

## Data Availability

The data that support the findings of this study are available from the corresponding author upon reasonable request.
